# Patterns of daily ambulatory activity and the onset of metabolic syndrome in middle-aged and older Japanese women: the Toon Health Study

**DOI:** 10.1265/ehpm.24-00313

**Published:** 2025-02-28

**Authors:** Naofumi Yamamoto, Koutatsu Maruyama, Isao Saito, Kiyohide Tomooka, Takeshi Tanigawa, Ryoichi Kawamura, Yasunori Takata, Haruhiko Osawa

**Affiliations:** 1 Faculty of Collaborative Regional Innovation, Ehime University, 3 Bunkyo-cho, Matsuyama, Ehime 790-8577, Japan; 2 Laboratory of Community Health and Nutrition, Special Course of Food and Health Science, Department of Bioscience, Graduate School of Agriculture, Ehime University, 3-5-7 Tarumi, Matsuyama, Ehime 790-8566, Japan; 3 Department of Public Health and Epidemiology, Faculty of Medicine, Oita University, 1-1 Idaigaoka, Hasama-machi, Yufu, Oita 879-5593, Japan; 4 Department of Public Health, Juntendo University Faculty of Medicine, 2-1-1 Hongo, Bunkyo-ku, Tokyo 113-8421, Japan; 5 Department of Public Health, Juntendo University Graduate School of Medicine, 2-1-1 Hongo, Bunkyo-ku, Tokyo 113-8421, Japan; 6 Department of Diabetes and Molecular Genetics, Ehime University Graduate School of Medicine, Shitsukawa, Toon, Ehime 791-0295, Japan

**Keywords:** Latent profile analysis, Longitudinal study, Physical activity, Obesity

## Abstract

**Background:**

This cohort study aimed to identify the accumulation patterns of objectively measured ambulatory activity (AA) variables in the middle-aged and older Japanese women and examine the relationship of these derivative patterns with onset of metabolic syndrome (MetS).

**Methods:**

A total of 794 women (mean age: 56.2 years) provided objectively assessed AA data using a uniaxial accelerometer. The number of steps, time accumulated in light-intensity AA (LIAA) and moderate-to-vigorous intensity AA (MVAA) and the ratio of MVAA to total AA (LIAA + MVAA) were calculated. Latent profile analysis was used to identify participant groups based on their distinct AA patterns. Logistic regression models were used to assess the association of groups with the onset of MetS after adjusting for age, sex, education, alcohol habit, smoking habit, energy intake, and the number of MetS components present at baseline.

**Results:**

Four distinct groups were identified: Group A had low levels of the AA variable; group B accumulated a certain number or more steps primarily through MVAA; group C accumulated a certain number or more steps primarily through LIAA; and group D had high level of the AA variables. Over the course of the 5-year follow-up period, 61 participants (7.7%) developed MetS. The multivariate-adjusted odds ratio (95% confidence interval) for onset of MetS in groups B, C, and D relative to group A were 0.416 (0.166–1.218), 0.451 (0.223–0.914), and 0.933 (0.365–2.382), respectively. Group C had a significantly lower odds ratio of MetS onset than group A.

**Conclusion:**

AA patterns accumulating a certain number or more steps, regardless of the intensity of AA, may help reduce the risk of MetS compared to inactive AA patterns.

## Graphical Abstract

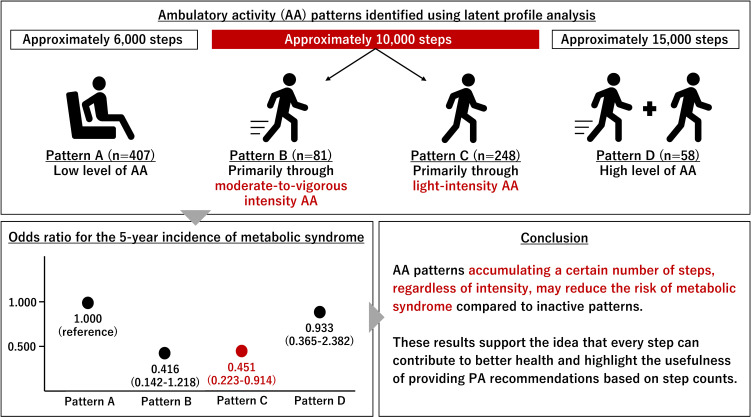

## Introduction

Metabolic syndrome (MetS) is defined as a cluster of conditions—including abdominal obesity, hypertension, dyslipidemia, and hyperglycemia—that increases the onset risk of various conditions, such as type 2 diabetes, cardiovascular disease, and cancer [1]. Previous studies have also suggested that MetS is more strongly associated with cardiovascular disease and death in women than in men [2]. Globally, the prevalence of MetS is increasing in both men and women [3], and in the Asia-Pacific region, >20% of adults are estimated to have MetS [4].

Physical activity (PA) is an important factor that can be modified to prevent MetS [5–7]. Most PA in daily life is accounted for by ambulatory activity (AA) such as walking and running [8, 9]. AA is an easy PA to practice and is considered the easiest method to go through daily life actively [10]. Previous study reported a negative association between the daily amount of AA and the time spent at different AA intensities and the onset of MetS [11]. However, the daily amount of AA, typically measured in step counts, results from AA of different intensities, which means that these AA variables are mutually related [12]. Little is known about the real-world accumulation patterns of AA and which patterns are associated with MetS. This information is considerably useful in the field of public health, especially to examine strategies that involve promoting PA to prevent MetS.

Latent profile analysis identifies the mutual relationship between input variables to create naturally occurring profiles, or typical patterns, of combinations of different variables in a heterogeneous population [13, 14]. In other words, the latent profile analysis approach is useful for enhancing the understanding of how mutual relationships—in particularly those that occur concomitantly between more than two variables—occur within-persons, and how these relationships are related in outcome variables [15, 16]. In recent years, latent profile analysis has been gaining attention in the field of PA as a useful statistical method to convert complex behavior into patterns [17, 18].

Recently, we identified AA patterns in the daily life of middle-aged and older Japanese people using latent profile analysis, and examined the relationship with MetS in cross-sectional study [19]. The results suggested that AA pattern involving a certain number or greater of steps accumulated through not only moderate-to-vigorous intensity AA (MVAA), but also light-intensity AA (LIAA) may help reduce the risk of MetS compared to inactive AA pattern [19]. However, while cross-sectional study may be susceptible to reverse causality, it was difficult to infer causal relationships. Therefore, in this study, we used a cohort study design to investigate the association of identified daily AA patterns by latent profile analysis with MetS onset among middle-aged and older Japanese women.

## Methods

### Study population

**Fig. 1:**
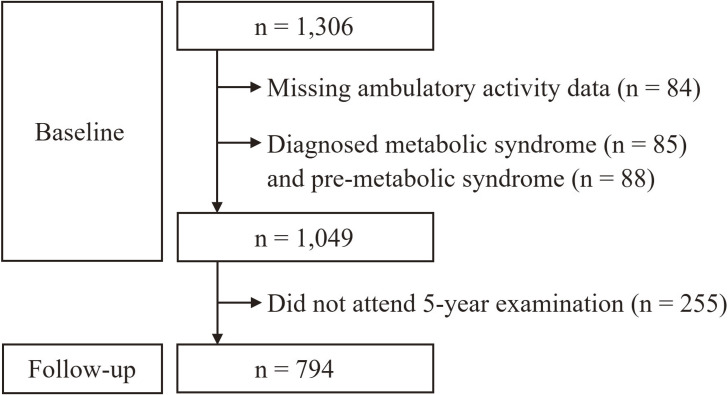
Flow diagram of participant selection in this study

This study was conducted as part of the Toon Health Study, a prospective cohort study that was planned to follow up the participants every 5 years in Toon City, Ehime Prefecture, Japan [11, 20]. The Toon Health Study commenced in 2009, with the aim to characterize risk factors for cardiovascular disease and its prevention in a community setting. In the Toon Health Study, we recruited participants from approximately 22,000 residents, aged 30–79 years, in Toon City using newspaper advertisements, posters, and invitations. In total, 1,306 women participated in the baseline survey conducted from 2009 to 2012. Of these, individuals with MetS (n = 85) and pre-MetS (n = 88), and those who had missing AA data (refusal to wear accelerometers, n = 63; wearing accelerometers for less than the required time/days, n = 21) were excluded. After excluding 255 individuals who did not undergo the 5-year examination, the remaining 794 were followed for 5 years. Therefore, the final analysis included 794 women, with a follow-up period of 5 years for all participants. The characteristics of the participants are presented in Table 1. The flow diagram of the participant selection process in this study is presented in Fig. 1. While our cohort included men, too few (n = 240) met the eligibility criteria, and therefore, they were excluded from the analysis.

**Table 1:** Characteristics of the study population at baseline

	**N = 794**	
Age (years)	56.2	(11.7)
Education (education past high school)	377	47.5
Smokers (n, %)	22	2.8
Drinkers (n, %)	278	35.0
Energy intake (kcal/day)	1861	(382)

Body mass index (kg/m^2^)	21.9	(2.7)
Waist circumference (cm)	79.7	(7.6)
Systolic blood pressure (mmHg)	122.6	(20.4)
Diastolic blood pressure (mmHg)	72.9	(11.5)
Fasting glucose (mg/dL)	90.9	(12.1)
High-density lipoprotein cholesterol (mg/dL)	65.5	(13.7)
Triglycerides (mg/dL)	92.6	(46.9)

Abdominal obesity (n, %)	28	3.5
Hypertension (n, %)	311	39.2
Hyperglycemia (n, %)	43	5.4
Dyslipidemia (n, %)	181	22.8
Having number of MetS components for diagnostic criteria (n, %)		
0	376	47.4
1	289	36.4
2	113	14.2
3	16	2.0

Steps (step/day)	8630	(3106)
LIAA (min/day)	72.0	(24.0)
MVAA (min/day)	21.2	(14.2)
Total AA (min/day)	93.3	(32.4)
MVAA/Total AA (min/min)	0.22	(0.10)

Data presented as mean (standard deviation) or n (%)

MetS, metabolic syndrome; LIAA, light-intensity ambulatory activity; MVAA, moderate- to vigorous-intensity ambulatory activity; AA, ambulatory activity

Written informed consent was given by all participants. The study protocol was approved by the Institutional Review Board, Ehime University Hospital (approval number: 170511).

### Ambulatory activity

Regarding AA, we evaluated the number of steps and period of activity according to intensity using a Lifecorder-Ex (LC, Suzuken Co., Ltd., Nagoya, Japan) with a uniaxial accelerometer [21]. This device detects acceleration signals ranging from 0.06 G to 1.94 G at 32 Hz. When the sensor detects three or more accelerations within 4 s, such activity is deemed as PA and classified into the LC’s unique activity intensity level of 1–9. Furthermore, when the level of acceleration is <0.06 G, the movement intensity will be 0. Moreover, when acceleration of ≥0.06 G is detected, which does not correspond to activity intensities 1–9, it will be perceived as micromovement and obtain an activity intensity value of 0.5. Thus, every 4 s, each activity will be classified and recorded into one of 11 levels (0, 0.5, and 1–9) of activity intensity. The activity intensity on the LC was reported to be closely approximated to the metabolic equivalents (METs) when walking and running [21]. Furthermore, based on previous research [21], the LC’s activity intensities of 1–3 and 4–9 were defined as LIAA and MVAA, respectively, where intensities of 1–3 correspond to less than 3 METs, and intensities of 4–9 correspond to 3 METs or more. The high level of measurement accuracy of the number of steps on the LC has been previously elucidated [22, 23].

The participants were instructed to wear the LC for 7 days continuously from waking up until bedtime, except when sleeping and during activities involving water (taking a bath and swimming). In the present study, an LC intensity of ≥0.5 for a total of ≥8 hours in 1 day, and for ≥4 days was defined as valid data [11, 19, 24] in which we calculated the mean number of steps, LIAA, MVAA, and total AA (LIAA + MVAA) per day for each individual.

### MetS components

Height was measured to the nearest 0.1 cm using a wall-mounted stadiometer. Body weight was measured to the nearest 0.1 kg using a digital scale without shoes. Body mass index was calculated from body weight and height (kg/m^2^). Waist circumference (WC) was measured three times to the nearest 0.1 cm using a calibrated measuring tape at the midpoint of the lower costal margin; the mean value was used in the analyses.

Blood pressure (BP) was measured twice using an automatic sphygmomanometer (BP-103iII; OMRON Colin Co., Tokyo, Japan) when the participants were seated after a rest of at least 5 minutes. The mean values of the two measurements were used for analysis.

Overnight fasting blood samples were collected from the antecubital vein into vacuum tubes containing a serum separator gel—to determine glucose levels and blood chemistry. The serum tube was centrifuged immediately at 3,000 g for 15 minutes, and the separated serum was sent to the laboratory for analysis. Enzymatic methods were used to measure serum levels of total cholesterol and triglycerides (TG). Low-density lipoprotein cholesterol and high-density lipoprotein cholesterol (HDL-C) were measured using the direct homogeneous method. Lipid measurements were standardized using the CDC NHLBI Lipids Standardization Program provided by the Centers for Disease Control and Prevention (Atlanta, GA, USA). Serum glucose was measured using the hexokinase method (Sysmex, Kobe, Japan) with an automatic analyzer (7600-D; Hitachi Co., Tokyo, Japan).

In this study, the Japanese criteria for MetS were used to establish the diagnosis of MetS and pre-MetS [25]. MetS was diagnosed when the participant had abdominal obesity (WC of ≥90 cm) in addition to ≥2 of the other components. Pre-MetS was diagnosed when the participant had abdominal obesity and one of the other components, including (1) dyslipidemia [TG ≥1.7 mmol/L and/or HDL-C level <1.0 mmol/L or specific treatment for these lipid abnormalities]; (2) BP ≥130/85 mmHg or on drug treatment; and (3) fasting glucose ≥6.1 mmol/L or on drug treatment. Ministry of Health, Labor, and Welfare of Japan considers MetS and pre-MetS as important targets to reduce the risk of MetS-related diseases [26]. Therefore, in line with previous studies [11, 19, 27–29], we analyzed AA pattern for participants in MetS and pre-MetS in a single group (MetS/pre-MetS).

### Covariates

Using a self-administered questionnaire, we surveyed smoking habits, alcohol consumption, and energy intake at baseline, and we surveyed the highest educational qualifications at follow-up. Individuals who responded that they presently smoked tobacco were considered smokers, and those who consumed ≥1 g of alcohol per week were considered drinkers. The usual consumption of food and beverage items were assessed by a food frequency questionnaire for which reasonable validity was observed in a previous study [30], and energy intake was estimated using the Standard Tables of Food Composition in Japan (2010) [31].

### Statistical analysis

As previous study [19], four variables were used in the latent profile analysis, namely the number of steps, LIAA, MVAA, and the ratio of MVAA to total AA. To characterize the AA patterns, we calculated and used the ratio of MVAA to total AA, which is proportionate of MVAA to daily total AA, in addition to three variables of AA measured using the LC (number of steps, LIAA, and MVAA). These variables were converted into z score for use in the analysis [19, 32]. To identify the latent profile, we implemented a continuous latent model with 2–5 profile solutions. To derive the optimal number of profiles, the Akaike Information Criterion (AIC), Bayesian Information Criterion (BIC), entropy, and group size of each profile was evaluated for each model from the two-profile model to a five-profile model. In addition, we performed a group interpretation [17] and selected the final model. Lower the AIC and BIC values, better the goodness-of-fit of the model. For entropy, a value of 0–1 was given, and values ≥0.8 were considered to indicate good profile classification. Group size was based on ≥5∼10% of participants belonging to each group of the total participant size [32, 33]. These fit statistics are commonly used in latent profile analysis [34]. For the latent profile analysis, we used Mplus (Version 8.5, Muthén & Muthén, Los Angeles, CA).

Categorical variables between AA patterns (groups) identified by latent profile analysis were compared using a chi-square test, and to compare continuous variables, a one-way analysis of variance was performed using the Bonferroni method for multiple comparisons. To examine the relationship between AA pattern and MetS onset, we conducted a logistic regression analysis using MetS as a dependent variable and AA patterns (groups) as independent variables. On the basis of the group with the lowest activity level, we calculated the odds ratio and the 95% confidence interval (95% CI) of the other groups. As confounder variables, we incorporated age (continuous variable), education (high school graduate or lower/education past high school), smoking habit (smoker/nonsmoker), drinking habit (drinker/nondrinker), energy intake (continuous variable), and the number of MetS component present at baseline (≥1/0) into the model. In the follow-up survey (5-year examination), those who corresponded to MetS or pre-MetS were defined as cases. The statistical analyses were performed using software SPSS version 28.0 (IBM Corp, Armonk, NY, USA), and *p* < 0.05 was considered statistically significant (two-tailed test).

## Results

The fit statistic of the latent profile model is presented in Table 2. The AIC and BIC decreased as the profile number increased in the two-to-five-profile models. In all models, a value of ≥0.8 was obtained for entropy. Regarding group size, all models included groups with more participants than the minimum recommended cutoff value (5%). However, the five-profile model included two relatively small groups (9.0 and 5.7%). Based on these fit statistics and the interpretability of the later-mentioned groups, we used the four-profile model as the optimal model in the present study.

**Table 2:** Fit indices of 2–5-profile latent models

	**2 Profiles**	**3 Profiles**	**4 Profiles**	**5 Profiles**
AIC	7959	7497	7170	6801
BIC	8019	7581	7278	6931
Entropy	0.894	0.834	0.877	0.879
Group size	618/176	405/291/98	407/248/81/58	310/275/92/72/45

AIC, Akaike information criterion; BIC, Bayesian information criteria

The AA characteristics of the four groups classified through latent profile analysis are presented in Table 3. The total AA was significantly high in descending order of groups A, B, C, and D; however, no significant difference observed in the number of steps between groups B and C. Group B had significantly higher MVAA than groups A and C, whereas group C had significantly higher LIAA than groups A and B.

**Table 3:** Ambulatory activity variables among the identified distinct groups at baseline

	**Group-A (n = 407)**		**Group-B (n = 81)**		**Group-C (n = 248)**		**Group-D (n = 58)**		***P***

	**mean**	**s.d.**	**mean**	**s.d.**	**mean**	**s.d.**	**mean**	**s.d.**	
Steps (step/day)	6278	1339^b,c,d^	10499	1668^a,d^	10250	1459^a,d^	15597	1739^a,b,c^	<0.001
LIAA (min/day)	56.7	12.7^b,c,d^	63.9	13.6^a,c,d^	90.6	16.5^a,b,d^	111.5	23.8^a,b,c^	<0.001
MVAA (min/day)	12.3	5.7^b,c,d^	40.8	8.8^a,c,d^	22.6	6.0^a,b,d^	51.3	16.2^a,b,c^	<0.001
Total AA (min/day)	69.0	14.7^b,c,d^	104.7	17.0^a,c,d^	113.1	17.4^a,b,d^	162.8	19.1^a,b,c^	<0.001
MVAA/Total AA (min/min)	0.18	0.07^b,c,d^	0.39	0.07^a,c,d^	0.20	0.05^a,b,d^	0.32	0.10^a,b,c^	<0.001

^a^p < 0.05 vs Group-A; ^b^p < 0.05 vs Group-B; ^c^p < 0.05 vs Group-C; ^d^p < 0.05 vs Group-D

LIAA, light-intensity ambulatory activity; MVAA, moderate- to vigorous-intensity ambulatory activity; AA, ambulatory activity

The baseline characteristics of the four groups are presented in Table 4. The proportion of drinkers was highest in group C. The fasting glucose levels in group D tended to be higher than those in other groups, while the HDL-C levels in group A tended to be lower compared to the other groups.

**Table 4:** Characteristics of participants in the identified distinct groups at baseline

	**Group-A (n = 407)**		**Group-B (n = 81)**		**Group-C (n = 248)**		**Group-D (n = 58)**		**p**
Age (years)	56.4	(12.4)	57.6	(11.4)	55.3	(11.2)	57.4	(10)	0.360
Education (education past high school)	195	47.9	40	49.4	114	46.0	28	48.3	0.942
Smokers (n, %)	13	3.2	1	1.2	5	2.0	3	5.2	0.432
Drinkers (n, %)	138	33.9	16	19.8	100	40.3	24	41.4	0.006
Energy intake (kcal/day)	1836	(383)	1853	(355)	1897	(392)	1901	(353)	0.198

Body mass index (kg/m^2^)	21.9	(2.6)	22.1	(2.2)	21.9	(2.8)	22.0	(3.1)	0.939
Waist circumference (cm)	80.2	(7.3)	79.4	(7.5)	79.2	(7.5)	79.3	(9.6)	0.382
Systolic blood pressure (mmHg)	121.9	(20.6)	126.0	(21.3)	122.1	(19.7)	125.0	(21.3)	0.292
Diastolic blood pressure (mmHg)	72.7	(11.6)	74.8	(11.0)	72.5	(11.1)	74.1	(12.4)	0.327
Fasting glucose (mg/dL)	90.8	(13.9)	91.7	(10.6)	89.9	(8.1)	94.6	(13.8)	0.059
High-density lipoprotein cholesterol (mg/dL)	64.0	(14)	67.1	(12.6)	66.5	(13.7)	69.3	(12.7)	0.009
Triglycerides (mg/dL)	96.2	(53.4)	91.9	(40.6)	88.3	(37.2)	87.0	(42.4)	0.148

Abdominal obesity (n, %)	10	2.5	3	3.7	10	4.0	5	8.6	0.112
Hypertension (n, %)	166	40.8	38	46.9	83	33.5	24	41.4	0.112
Hyperglycemia (n, %)	23	5.7	5	6.2	9	3.6	6	10.3	0.219
Dyslipidemia (n, %)	99	24.3	20	24.7	48	19.4	14	24.1	0.487
Having number of MetS components for diagnostic criteria (n, %)									
0	190	46.7	32	39.5	132	53.2	22	37.9	0.137
1	147	36.1	32	39.5	86	34.7	24	41.4	
2	59	14.5	17	21.0	26	10.5	11	19.0	
3	11	2.7	0	0.0	4	1.6	1	1.7	

Data presented as mean (standard deviation) or n (%). MetS, metabolic syndrome

Over the 5-years follow-up period, 61 participants (7.7%) developed MetS. The crude and multivariate adjustment odds ratios for MetS onset according to the four groups have been presented in Table 5. The multivariate-adjusted odds ratio (95% CI) of groups B, C, and D to groups A were 0.416 (0.142–1.218), 0.451 (0.223–0.914), and 0.933 (0.365–2.382), respectively, with a significantly lower value obtained for groups C. In a sensitivity analysis excluding individuals with each MetS component at baseline, groups B and C consistently showed a lower odds ratio for MetS onset than group A (Table 6). In a latent profile analysis, each individual was assigned to a class based on their highest posterior probability. To increase statistical power, we conducted an analysis by reassigning five individuals who had relatively high posterior probabilities (>0.30) for Group B to this group. The multivariate-adjusted odds ratios (95% CI) of Groups B (n = 86), C (n = 244), and D (n = 58) compared to Group A (n = 406) were 0.378 (0.129–1.105), 0.460 (0.227–0.934), and 0.927 (0.363–2.368), respectively. As a result, we observed that the upper bound of the 95% CI for Group B decreased, approaching closer to 1.000.

**Table 5:** OR and 95% CI for MetS onset in the identified distinct groups

	**n**	**Case**	**Crude OR (95% CI)**		**Multivariable adjusted OR^a^ (95% CI)**	
Group-A	407	40	1.000	(reference)	1.000	(reference)
Group-B	81	4	0.477	(0.166–1.371)	0.416	(0.142–1.218)
Group-C	248	11	0.426	(0.214–0.846)	0.451	(0.223–0.914)
Group-D	58	6	1.059	(0.428–2.619)	0.933	(0.365–2.382)

^a^Adjusted for age (continuous variable), education (high school graduate or lower/education past high school), smoking habit (smoker/nonsmoker), drinking habit (drinker/nondrinker), energy intake (continuous variable), and the number of MetS components present at baseline (≥1/0).

OR, odds ratio; CI, confidence interval; MetS, metabolic syndrome

**Table 6:** OR and 95% CI for MetS onset in baseline component-free participants

	**Group-A**		**Group-B**		**Group-C**		**Group-D**	
Participants excluding those with abdominal obesity at baseline								
n/case	397/38		78/3		228/10		49/4	
Crude OR (95% CI)	1.000	(reference)	0.378	(0.114–1.256)	0.414	(0.202–0.848)	0.771	(0.264–2.254)
Multivariable adjusted OR^a^ (95% CI)	1.000	(reference)	0.329	(0.097–1.116)	0.456	(0.218–0.953)	0.712	(0.236–2.150)

Participants excluding those with hypertension at baseline								
n/case	241/12		43/1		165/4		34/4	
Crude OR (95% CI)	1.000	(reference)	0.454	(0.058–3.588)	0.474	(0.150–1.496)	2.544	(0.771–8.396)
Multivariable adjusted OR^a^ (95% CI)	1.000	(reference)	0.382	(0.046–3.183)	0.467	(0.142–1.533)	1.906	(0.532–6.820)

Participants excluding those with hyperglycemia at baseline								
n/case	384/34		76/4		239/10		52/6	
Crude OR (95% CI)	1.000	(reference)	0.572	(0.197–1.662)	0.450	(0.218–0.928)	1.343	(0.535–3.372)
Multivariable adjusted OR^a^ (95% CI)	1.000	(reference)	0.503	(0.169–1.495)	0.475	(0.226–0.999)	1.216	(0.465–3.182)

Participants excluding those with dyslipidemia at baseline								
n/case	308/22		61/2		200/10		44/3	
Crude OR (95% CI)	1.00	(reference)	0.441	(0.101–1.925)	0.684	(0.317–1.477)	0.951	(0.273–3.320)
Multivariable adjusted OR^a^ (95% CI)	1.000	(reference)	0.350	(0.078–1.572)	0.688	(0.308–1.537)	0.741	(0.204–2.693)

^a^Adjusted for age (continuous variable), education (high school graduate or lower/education past high school), smoking habit (smoker/nonsmoker), drinking habit (drinker/nondrinker), energy intake (continuous variable), and the number of MetS components present at baseline (≥1/0).

OR, odds ratio; CI, confidence interval; MetS, metabolic syndrome

## Discussion

This study adopted a latent model with four-profile solutions as the optimum model. Group A was a “low-level AA pattern,” group B was a “pattern in which a certain level of steps [35] was accumulated mainly by MVAA,” group C was a “pattern in which a certain level of steps [35] was accumulated by LIAA,” and group D was a “high-level AA pattern.” In our previous study that attempted to identify AA patterns in middle-aged and older Japanese through latent profile analysis, AA activities in daily life could be classified into four categories: “low-level AA patterns,” “AA patterns in which a certain level of number of steps is accumulated mainly by MVAA,” “AA patterns in which a certain level of number of steps is accumulated by both MVAA and LIAA,” and “high-level AA patterns.” [19] The results of this study were generally consistent with the results of previous study [19]. The participants of this study are a subgroup of the previous study’s participants [19], excluding those with MetS and men. The similar results from different participants reinforce the possibility of such AA patterns in the real world. Based on previous research [19], group A was interpreted as an “insufficient” pattern, group B as an “active couch potato” pattern [36], group C as a “move more and sit less” pattern [37], and group D as a “Busy Bee” pattern [38] of PA. In the five-profile model, there were two groups with high AA levels, but the number of individuals belonging to these profiles was relatively small (9.0% and 5.7%) [34]. The low-level AA pattern, the AA pattern accumulating a certain level of steps mainly by MVAA, and the AA pattern accumulating a certain level of steps mainly by LIAA were similarly present in the five-profile model.

In this study, the odds ratio for the onset of MetS was similarly low in groups B and C and observed to be significantly lower than the reference in group C. Many previous studies have examined the relationship between moderate-to-vigorous intensity PA and the onset of MetS, noting a significant negative dose-response relationship between the two [5, 6]. In recent years, the health benefits of light-intensity PA have also received attention [39]. Since light-intensity PA is a major determinant of daily PA-induced energy expenditure [40], it can be inferred that its level may be closely related to the onset of abdominal obesity, a central component of MetS. In addition, light-intensity PA has been reported to be negatively associated with cardiometabolic risk factors such as WC and TG, independently of moderate-to-vigorous intensity PA [39]. Furthermore, a negative association exists between light-intensity PA and daily sedentary time [19]. Previous studies have also reported that longer sedentary time may increase the risk of developing MetS [7]. Compared to group B, group C has a shorter MVAA time of about 20 minutes but a LIAA time of about 30 minutes longer and a total AA time of about 10 minutes longer. This additional effect of LIAA likely contributes to the lower odds ratio for MetS onset in group C, similar to that of group B.

The Japanese PA guidelines recommend intentional PA, such as exercise, as well as activities of daily living, such as non-exercise activity, as specific practices to increase daily PA [41]. The number of steps is an easy-to-understand indicator of the daily amount of PA and is a simple and feasible measure for monitoring and promoting PA as fitness trackers and mobile devices have increased in popularity [42]. For this reason, it has rapidly gained attention in the field of PA in recent years, and discussion is ongoing about showing the recommended amount of PA for health promotion using the measure of steps [42]. Therefore, evidence has accumulated on the number of steps and various health outcomes [43]. In general, LIAA is thought to occur and accumulate mainly in activities of daily living, such as housework and work, while MVAA is thought to occur and accumulate mainly in intentional PA, such as exercise [44, 45]. The notable points of this study are: 1) it showed that there are patterns in the real world in which a certain level of steps is accumulated mainly by MVAA or mainly by LIAA as recommended by the Japanese PA guidelines [41] and 2) it suggested that there is no major difference in preventing the onset of MetS between the different patterns of step accumulation. This information supports the assertion that any increase in daily step count should be considered for inclusion in public health guidelines [43], and may further emphasize the importance of daily step monitoring for public health. In this study, we believe that through person-centered analysis of findings obtained by variable-centered analysis to date [11], we were able to provide additional useful information to be implemented in public health activities to prevent MetS.

The main strength of this study is the objective measurement of daily AA using the accelerometer. Due to the difficulty in remembering such relatively low-intensity and intermittently occurring PAs, self-reported AA measurement may not be reliable and valid [46]. The objective methods guarantee more accuracy of AA assessment than self-reported methods. However, this study also has several limitations. First, the participants in this study were limited to middle-aged and older Japanese women. Walking speed declines with age [47], and Japanese women traditionally spend more time on housekeeping than men, leading to a higher proportion of low-intensity PA relative to total activity time [44, 45]. Therefore, even if men and women have the same number of steps, their walking patterns may differ. As a result, the findings of this study may not be generalizable to other age groups, sexes, or populations in different countries. Future research should explore the applicability of these findings across various age groups, sexes, and cultural contexts. Second, the participants in this study were volunteers, which may have introduced selection bias. According to the National Health and Nutrition Survey in Japan, the average number of steps taken by the participants in this study was slightly higher than the national average for Japanese women [48]. This suggests that the participants were more likely to engage in a healthy lifestyle compared to the general population. Therefore, the findings of this study may not necessarily be applicable to the general population of middle-aged and older women. Future studies should consider examining a more representative sample to enhance the generalizability of the results. Third, in this study, AA was assessed only once at baseline. An 8-year follow-up study among Japanese individuals demonstrated that AA, as assessed by step count, maintained high stability over time, preserving its relative ranking within the population [49]. Thus, we believe that baseline AA measurements generally reflect AA levels during the follow-up period. However, since changes in AA during the follow-up period can lead to an underestimation of the true association between AA and the onset of MetS [50], the relationship observed in this study may have been underestimated. To assess more accurately the association between AA patterns and MetS onset, additional studies with designs that include multiple measurements of AA are warranted. Fourth, in this study, group B did not reach statistical significance, although it showed a low odds ratio for the MetS onset. No certain trend was observed for group D. We speculate that the lack of statistical power for group B is due to the small number of MetS onset and group D due to the small group size. Future studies with larger sample sizes are needed to confirm these findings. Finally, the follow-up period in this study was relatively short at 5 years. A 5-year follow-up may miss late-onset cases, potentially reducing the statistical power. A longer follow-up would allow for a more detailed examination of the onset of MetS over time and help mitigate the risk of reverse causation. We plan to extend the follow-up period to assess the association more accurately between AA patterns and MetS risk.

## Conclusion

In the present study, we identified AA patterns in the daily life of middle-aged and older Japanese women, and examined the relationship with onset of MetS. The AA patterns of middle-aged and older Japanese women can be classified into four patterns. Our findings suggest that AA patterns involving the accumulation of a certain number of steps, regardless of the intensity of AA, may help reduce the risk of MetS compared to inactive AA patterns. These results support the idea that any step may contribute to better health and highlight the usefulness of providing PA recommendations based on step counts.

## Abbreviations

AA: Ambulatory activity
AIC: Akaike Information Criterion
BIC: Bayesian Information Criterion
BP: Blood pressure
HDL-C: high-density lipoprotein cholesterol
LC: Lifecorder
LIAA: light-intensity ambulatory activity
METs: Metabolic equivalents
MetS: Metabolic syndrome
MVAA: Moderate-to-vigorous intensity ambulatory activity
PA: Physical activity
TG: Triglycerides
WC: Waist circumference
95% CI: 95% confidence interval

## Declarations

Written informed consent was given by all participants. The study protocol was approved by the Institutional Review Board, Ehime University Hospital (approval number: 170511).

### Availability of data and material

Available upon a reasonable request.

## References

[1] Cornier MA, Dabelea D, Hernandez TL, Lindstrom RC, Steig AJ, Stob NR, Van Pelt RE, Wang H, Eckel RH. The metabolic syndrome. Endocr Rev. 2008;29:777–822. 18971485 10.1210/er.2008-0024 PMC5393149

[2] Gami AS, Witt BJ, Howard DE, Erwin PJ, Gami LA, Somers VK, Montori VM. Metabolic syndrome and risk of incident cardiovascular events and death: a systematic review and meta-analysis of longitudinal studies. J Am Coll Cardiol. 2007;49:403–14. 17258085 10.1016/j.jacc.2006.09.032

[3] Saklayen MG. The Global Epidemic of the Metabolic Syndrome. Curr Hypertens Rep. 2018;20:12. 29480368 10.1007/s11906-018-0812-z PMC5866840

[4] Ranasinghe P, Mathangasinghe Y, Jayawardena R, Hills AP, Misra A. Prevalence and trends of metabolic syndrome among adults in the Asia-Pacific region: a systematic review. BMC Public Health. 2017;17:101. 28109251 10.1186/s12889-017-4041-1 PMC5251315

[5] Zhang D, Liu X, Liu Y, Sun X, Wang B, Ren Y, Zhao Y, Zhou J, Han C, Yin L, Zhao J, Shi Y, Zhang M, Hu D. Leisure-time physical activity and incident metabolic syndrome: a systematic review and dose-response meta-analysis of cohort studies. Metabolism. 2017;75:36–44. 28927737 10.1016/j.metabol.2017.08.001

[6] Amirfaiz S, Shahril MR. Objectively measured physical activity, sedentary behavior, and metabolic syndrome in adults: systematic review of observational evidence. Metab Syndr Relat Disord. 2019;17:1–21. 30272527 10.1089/met.2018.0032

[7] Wu J, Zhang H, Yang L, Shao J, Chen D, Cui N, Tang L, Fu Y, Xue E, Lai C, Ye Z. Sedentary time and the risk of metabolic syndrome: a systematic review and dose-response meta-analysis. Obes Rev. 2022;23:e13510. 36261077 10.1111/obr.13510

[8] Bijnen FC, Feskens EJ, Caspersen CJ, Mosterd WL, Kromhout D. Age, period, and cohort effects on physical activity among elderly men during 10 years of follow-up: the Zutphen Elderly Study. J Gerontol A Biol Sci Med Sci. 1998;53:M235–41. 9597057 10.1093/gerona/53a.3.m235

[9] Tanaka C, Fujiwara Y, Sakurai R, Fukaya T, Yasunaga M, Tanaka S. Locomotive and non-locomotive activities evaluated with a triaxial accelerometer in adults and elderly individuals. Aging Clin Exp Res. 2013;25:637–43. 24170329 10.1007/s40520-013-0163-1

[10] National Health Service. Walking for health. https://www.nhs.uk/Live-well/exercise/running-and-aerobic-exercises/walking-for-health/. Accessed Sep 19, 2024.

[11] Yamamoto N, Maruyama K, Saito I, Tomooka K, Tanigawa T, Kawamura R, Takata Y, Osawa H. Prospective association of daily ambulatory activity with metabolic syndrome in middle-aged and older Japanese adults: the Toon Health Study. Int J Obes (Lond). 2024;48:733–40. 38307954 10.1038/s41366-024-01483-w

[12] Thompson D, Batterham AM. Towards integrated physical activity profiling. PLoS One. 2013;8:e56427. 23437131 10.1371/journal.pone.0056427 PMC3577906

[13] Marsh HW, Luedtke O, Trautwein U, Morin A. Classical latent profile analysis of academic self-concept dimensions: Synergy of person- and variable centered approaches to theoretical models of self-concept. Struct Equ Modeling. 2009;16:191–225.

[14] Pastor DA, Barron KE, Miller BJ, Davis L. A latent profile analysis of college students’ achievement goal orientation. Contemp Educ Psychol. 2007;32:8–47.

[15] Ekblom-Bak E, Stenling A, Salier Eriksson J, Hemmingsson E, Kallings LV, Andersson G, Wallin P, Ekblom Ö, Ekblom B, Lindwall M. Latent profile analysis patterns of exercise, sitting and fitness in adults - Associations with metabolic risk factors, perceived health, and perceived symptoms. PLoS One. 2020;15:e0232210. 32330191 10.1371/journal.pone.0232210 PMC7182226

[16] Thogersen-Ntoumani C, Black J, Lindwall M, Whittaker A, Balanos GM. Presenteeism, stress resilience, and physical activity in older manual workers: a person-centered analysis. Eur J Ageing. 2017;14:385–96. 29180944 10.1007/s10433-017-0418-3 PMC5684036

[17] Miranda VPN, Coimbra DR, Bastos RR, Miranda Júnior MV, Amorim PRDS. Use of latent class analysis as a method of assessing the physical activity level, sedentary behavior and nutritional habit in the adolescents’ lifestyle: A scoping review. PLoS One. 2021;16:e0256069. 34411143 10.1371/journal.pone.0256069 PMC8376087

[18] Kebede M, Howard AG, Ren Y, Anuskiewicz B, Di C, Troester MA, Evenson KR. A systematic scoping review of latent class analysis applied to accelerometry-assessed physical activity and sedentary behavior. PLoS One. 2024;19:e0283884. 38252639 10.1371/journal.pone.0283884 PMC10802947

[19] Yamamoto N, Maruyama K, Saito I, Tomooka K, Tanigawa T, Kawamura R, Takata Y, Osawa H. Latent profile analysis approach to the relationship between daily ambulatory activity patterns and metabolic syndrome in middle-aged and elderly Japanese individuals: The Toon Health Study. Environ Health Prev Med. 2023;28:57. 37766543 10.1265/ehpm.23-00110 PMC10569967

[20] Saito I, Maruyama K, Kato T, Takata Y, Tomooka K, Kawamura R, Osawa H, Tanigawa T. Role of insulin resistance in the association between resting heart rate and type 2 diabetes: A prospective study. J Diabetes Complications. 2022;36:108319. 36279707 10.1016/j.jdiacomp.2022.108319

[21] Kumahara H, Schutz Y, Ayabe M, Yoshioka M, Yoshitake Y, Shindo M, Ishii K, Tanaka H. The use of uniaxial accelerometry for the assessment of physical-activity-related energy expenditure: a validation study against whole-body indirect calorimetry. Br J Nutr. 2004;91:235–43. 14756909 10.1079/BJN20031033

[22] Crouter SE, Schneider PL, Karabulut M, Bassett DR Jr. Validity of 10 electronic pedometers for measuring steps, distance, and energy cost. Med Sci Sports Exerc. 2003;35:1455–60. 12900704 10.1249/01.MSS.0000078932.61440.A2

[23] Schneider PL, Crouter S, Bassett DR. Pedometer measures of free-living physical activity: comparison of 13 models. Med Sci Sports Exerc. 2004;36:331–5. 14767259 10.1249/01.MSS.0000113486.60548.E9

[24] Nishida Y, Higaki Y, Taguchi N, Hara M, Nakamura K, Nanri H, Imaizumi T, Sakamoto T, Shimanoe C, Horita M, Shinchi K, Tanaka K. Intensity-specific and modified effects of physical activity on serum adiponectin in a middle-aged population. J Endocr Soc. 2018;3:13–26. 30560225 10.1210/js.2018-00255 PMC6293231

[25] Committee to Evaluate Diagnostic Standards for Metabolic Syndrome Definition and the diagnostic standard for metabolic syndrome. Nihon Naika Gakkai Zasshi. 2005;94:794–809 (in Japanese). 15865013

[26] Yamagishi K, Iso H. The criteria for metabolic syndrome and the national health screening and education system in Japan. Epidemiol Health. 2017;39:e2017003. 28092931 10.4178/epih.e2017003 PMC5343105

[27] Honda T, Chen S, Kishimoto H, Narazaki K, Kumagai S. Identifying associations between sedentary time and cardio-metabolic risk factors in working adults using objective and subjective measures: a cross-sectional analysis. BMC Public Health. 2014;14:1307. 25526746 10.1186/1471-2458-14-1307 PMC4302076

[28] Kim J, Tanabe K, Yokoyama N, Zempo H, Kuno S. Association between physical activity and metabolic syndrome in middle-aged Japanese: a cross-sectional study. BMC Public Health. 2011;11:624. 21819591 10.1186/1471-2458-11-624 PMC3199599

[29] Kudo N, Nishide R, Mizutani M, Ogawa S, Tanimura S. Association between the type of physical activity and metabolic syndrome in middle-aged and older adult residents of a semi-mountainous area in Japan. Environ Health Prev Med. 2021;26:46. 33838647 10.1186/s12199-021-00949-x PMC8035718

[30] Takahashi K, Yoshimura Y, Kaimoto T, Kunii D, Komatsu T, Yamamoto S. Validation of food frequency questionnaire based on food groups for estimating individual nutrient intake. Jpn J Nutr. 2001;59:221–32 (in Japanese).

[31] Report of the Subdivision on Resources the Council for Science and Technology Ministry of Education, Culture, Sports, Science and Technology, Japan. Standard Tables of Food Composition in Japan 2010. http://www.mext.go.jp/b_menu/shingi/gijyutu/gijyutu3/houkoku/1298713.htm. Accessed Sep 19, 2024.

[32] von Rosen P, Dohrn IM, Hagströmer M. Latent profile analysis of physical activity and sedentary behavior with mortality risk: A 15-year follow-up. Scand J Med Sci Sports. 2020;30:1949–56. 32615651 10.1111/sms.13761

[33] Verswijveren SJJM, Lamb KE, Leech RM, Salmon JO, Timperio A, Telford RM, McNarry MA, Mackintosh KA, Daly RM, Dunstan DW, Hume C, Cerin E, Olive LS, Ridgers ND. Activity Accumulation and Cardiometabolic Risk in Youth: A Latent Profile Approach. Med Sci Sports Exerc. 2020;52:1502–10. 31977636 10.1249/MSS.0000000000002275

[34] Aflaki K, Vigod S, Ray JG. Part II: a step-by-step guide to latent class analysis. J Clin Epidemiol. 2022;148:170–3. 35662622 10.1016/j.jclinepi.2022.05.009

[35] Cao ZB, Oh T, Miyatake N, Tsushita K, Higuchi M, Tabata I. Steps per day required for meeting physical activity guidelines in Japanese adults. J Phys Act Health. 2014;11:1367–72. 24366861 10.1123/jpah.2012-0333

[36] Owen N, Healy GN, Matthews CE, Dunstan DW. Too much sitting: the population health science of sedentary behavior. Exerc Sport Sci Rev. 2010;38:105–13. 20577058 10.1097/JES.0b013e3181e373a2 PMC3404815

[37] Singh R, Pattisapu A, Emery MS. US Physical Activity Guidelines: Current state, impact and future directions. Trends Cardiovasc Med. 2020;30:407–12. 31677904 10.1016/j.tcm.2019.10.002

[38] Marschollek M. Clustering physical activity phenotypes using the ATLAS index on accelerometric data from an epidemiologic cohort study. Stud Health Technol Inform. 2014;205:763–7. 25160290

[39] Amagasa S, Machida M, Fukushima N, Kikuchi H, Takamiya T, Odagiri Y, Inoue S. Is objectively measured light-intensity physical activity associated with health outcomes after adjustment for moderate-to-vigorous physical activity in adults? A systematic review. Int J Behav Nutr Phys Act. 2018;15:65. 29986718 10.1186/s12966-018-0695-z PMC6038338

[40] Lindsay T, Wijndaele K, Westgate K, Dempsey P, Strain T, De Lucia Rolfe E, Forouhi NG, Griffin S, Wareham NJ, Brage S. Joint associations between objectively measured physical activity volume and intensity with body fatness: the Fenland study. Int J Obes (Lond). 2022;46:169–77. 34593963 10.1038/s41366-021-00970-8 PMC8748201

[41] Ministry of Health, Labour and Welfare. Active guide: Japanese official physical activity guidelines for health promotion. https://www.nibiohn.go.jp/eiken/info/pdf/active2013-e.pdf. Accessed Sep 19, 2024.

[42] Kraus WE, Janz KF, Powell KE, Campbell WW, Jakicic JM, Troiano RP, Sprow K, Torres A, Piercy KL; 2018 PHYSICAL ACTIVITY GUIDELINES ADVISORY COMMITTEE. Daily step counts for measuring physical activity exposure and its relation to health. Med Sci Sports Exerc. 2019;51:1206–12. 31095077 10.1249/MSS.0000000000001932 PMC6527133

[43] Ao Z, He H, Shi H, Liu H. Step count and multiple health outcomes: An umbrella review. J Evid Based Med. 2024;17:278–95. 38566344 10.1111/jebm.12596

[44] Aoyagi Y, Shephard RJ. Habitual physical activity and health in the elderly: the Nakanojo Study. Geriatr Gerontol Int. 2010;10 Suppl 1:S236–43. 20590838 10.1111/j.1447-0594.2010.00589.x

[45] Amagasa S, Fukushima N, Kikuchi H, Takamiya T, Oka K, Inoue S. Light and sporadic physical activity overlooked by current guidelines makes older women more active than older men. Int J Behav Nutr Phys Act. 2017;14:59. 28464833 10.1186/s12966-017-0519-6 PMC5414194

[46] Ara I, Aparicio-Ugarriza R, Morales-Barco D, Nascimento de Souza W, Mata E, González-Gross M. Physical activity assessment in the general population; validated self-report methods. Nutr Hosp. 2015;31(Suppl 3):211–8. 25719788 10.3305/nh.2015.31.sup3.8768

[47] Ayabe M, Yahiro T, Yoshioka M, Higuchi H, Higaki Y, Tanaka H. Objectively measured age-related changes in the intensity distribution of daily physical activity in adults. J Phys Act Health. 2009;6:419–25. 19842455 10.1123/jpah.6.4.419

[48] Ministry of Health, Labour and Welfare of Japan. The National Health and Nutrition Survey in Japan, 2019. https://www.mhlw.go.jp/content/001066903.pdf. Accessed Sep 19, 2024.

[49] Yamamoto N, Shimada M, Nakagawa N, Sawada SS, Nishimuta M, Kimura Y, Ohashi M, Asai H, Miyazaki H, Lee IM, Blair SN, Yoshitake Y. Tracking of pedometer-determined physical activity in healthy elderly Japanese people. J Phys Act Health. 2015;12:1421–9. 25599246 10.1123/jpah.2014-0450

[50] Jebb SA, Moore MS. Contribution of a sedentary lifestyle and inactivity to the etiology of overweight and obesity: current evidence and research issues. Med Sci Sports Exerc. 1999;31(11 Suppl):S534–41. 10593524 10.1097/00005768-199911001-00008

